# The Impact of Surgical Conization of the Cervix and Loop Electrosurgical Excision Procedure on Female Sexual Function

**DOI:** 10.3390/cancers17061033

**Published:** 2025-03-20

**Authors:** Paweł Bartnik, Joanna Kacperczyk-Bartnik, Anna Różańska-Walędziak, Andrzej Wróbel, Christopher Kobierzycki, Krzysztof Czajkowski, Ewa Romejko-Wolniewicz

**Affiliations:** 1 II Department of Obstetrics and Gynaecology, Medical University of Warsaw, 02-091 Warsaw, Poland; 2 Department of Human Physiology and Patophysiology, Faculty of Medicine, Collegium Medicum, Cardinal Stefan Wyszynski University in Warsaw, 01-938 Warsaw, Poland; 3 Second Department of Gynecology, Medical University of Lublin, 20-090 Lublin, Poland; 4 Division of Histology and Embryology, Department of Human Morphology and Embryology, Wroclaw Medical University, 50-368 Wroclaw, Poland; 5 Department of Gynecology and Obstetrics, Poviat Hospital, 56-400 Olesnica, Poland

**Keywords:** cervical cancer, cervical dysplasia, conization, sexual health

## Abstract

**Objectives**: The aim of the study was to analyze and compare the possible effect of cervical conization and the loop electrosurgical excision procedure (LEEP) on female sexual function up to one year after intervention, as existing studies provide incoherent results. **Methods**: This prospective cohort study enrolled patients who underwent either LEEP (n = 35) or surgical conization of the cervix (n = 44). Patients completed the questionnaire before the intervention and at three, six, and twelve months after the end of the postoperative period. The questionnaire included the Polish version of the Female Sexual Function Index (FSFI) and the European Organization for Research and Treatment of Cancer Quality of Life Questionnaire of Cancer Patients with the module Cervix-24. **Results**: In the LEEP group, significant deterioration was observed in the FSFI orgasm subscale after three and six months in comparison to the baseline (3.98 ± 2.08 vs. 3.19 ± 2.29 vs. 3.09 ± 2.24; *p* < 0.02). The difference in the orgasm subscale compared to the baseline score was not reported after twelve months of follow-up. In the surgical conization group, significant deterioration was observed in the general FSFI score between the baseline and three months after (22.37 ± 12.38 vs. 20.82 ± 12.02; *p* < 0.003) and in the arousal subscale between the baseline and three months after (3.69 ± 2.14 vs. 3.01 ± 2.02; *p* < 0.001). In the orgasm subscale, there was a significant improvement between three and twelve months of observation (3.05 ± 2.22 vs. 3.63 ± 2.29; *p* < 0.003). A significant deterioration was observed in the sexual activity subscale of the EORTC QLQ-C30 + CX24 between baseline and after three months (49.42 ± 36.12 vs. 39.09 ± 36.81; *p* < 0.03). All reported deteriorations had a tendency to resolve within twelve months of observation. **Conclusions**: Both LEEP and surgical conization of the cervix seem to have a mild, transient negative impact on female sexual function, which normalizes one year after the procedure. Long-term consequences of both procedures are similar. Further research with larger sample sizes is necessary to confirm these findings.

## Simple Summary

This study was proposed to assess the potential association between two different types of treatment for cervical precancer or cancer lesions and female sexual function within one year after intervention. Forty-four women who had surgical conization and thirty-five women who underwent loop electrosurgical excision procedures were enrolled in this prospective cohort study. Patients completed questionnaires to compare their outcomes three, six, and twelve months after the treatment. The results showed that women in both studied groups developed mild, transient deteriorations in sexual function, which resolved spontaneously within one year of observation.

## 1. Introduction

Cervical cancer is a gynecological malignancy characterized by well-established methods of prevention. They include both primary methods, such as human papillomavirus (HPV) vaccinations, and secondary ones, such as Pap smears and HPV tests [1]. The use of secondary methods is aimed at the early detection of the disease at the pre-malignant level or in the early stage when definitive and minimally invasive surgical treatment is available.

Cervical intraepithelial neoplasia (CIN) abnormalities are precancerous changes in the cervix of the uterus. The conservative classification of CINs distinguishes three levels of increasing cancerous potential [2]. In most histologically verified cases of CIN 2 and 3, the treatment consists of either a loop electrosurgical excision procedure (LEEP) or surgical conization of the cervix [3]. Both procedures can also be optionally used in selected cases of cervical cancer limited to the FIGO IA1 and IA2 stages.

Different methods of cervical cancer treatment have multiple possible effects on the patient’s sexual function. Chemoradiotherapy treatment has a severe negative effect on female sexuality [4]. Regarding surgical procedures, most of the available studies focus on the effects of radical hysterectomy. The vast majority of publications prove it has a generally negative impact on female sexual function [5,6,7]. Even though procedures such as LEEP and surgical conization are more frequently performed, studies analyzing the possible effect of these procedures on female sexuality are sparse and have conflicting results [8,9,10,11,12,13,14,15,16,17].

This study aimed to analyze and compare the possible effect of cervical conization and LEEP on female sexual function up to one year after intervention.

## 2. Materials and Methods

This was a prospective cohort study of patients who underwent either LEEP or surgical conization of the cervix at a tertiary referral center. Patients underwent the aforementioned procedures between January 2018 and May 2019.

The inclusion criteria for the study were patients diagnosed with HPV-related cervical dysplasia (LGSIL or HGSIL) or cervical cancer who were scheduled for surgical treatment, either LEEP or surgical conization. Only patients who provided informed consent to participate in the study were included. Both sexually active and non-sexually active women were included, though a sub-group analysis was performed on sexually active women to assess the impact of surgery on sexual function. Patients who had undergone adjuvant radiation therapy or hormonal treatment were excluded to avoid confounding effects on sexual function, as these treatments are known to influence sexual health. Women who were pregnant or had been pregnant within the last 12 months were excluded, as pregnancy could confound the sexual health outcomes.

Patients were diagnosed and managed in line with the American Society for Colposcopy and Cervical Pathology (ASCCP) guidelines [3]. Patients were qualified for either LEEP or surgical conization of the cervix based on the localization of pathological changes (whether they affected the canal of the cervix or not), the age of the patient, and the individual anatomy of the cervix. All patients had high-grade cervical dysplasia (CIN2 or CIN3) or micro-invasive cervical cancer of FIGO stage IA1 histologically confirmed (one patient without the need for further treatment). LEEP was selected for smaller, well-defined lesions confined to the cervix, whereas cold knife conization was more commonly chosen for larger or more extensive lesions. For patients who have had prior cervical surgeries, cold knife conization was preferred due to its ability to provide larger, more accurate tissue samples. Individual preferences of the patient, including the potential risks and benefits of each procedure, were also considered.

Before the recruitment for the study, each patient received full information about the study protocol, and all patients’ questions were properly addressed. Each patient, both in the surgical conization group and the LEEP group, gave written consent for participation in the study. The protocol of the study was submitted for review and received an exemption from the Bioethics Committee of the Medical University of Warsaw (no. AKBE/31/2019).

At the time of the recruitment, patients were asked to fill in the baseline questionnaire. In the follow-up, they were asked to complete questionnaires at three, six, and twelve months after the end of the postoperative period. In the group of patients who underwent surgical conization, the end of the postoperative period was defined as six weeks after the surgery, when the Sturmdorf suture was removed. In the group of patients who underwent LEEP, it was defined as three weeks after the intervention. Cold knife conization typically involves a more extensive excision of tissue compared to LEEP, which is a less invasive procedure. As a result, patients who underwent cold knife conization generally required a longer recovery time, which in turn affected the timing of their postoperative assessments.

The questionnaires completed by the patients consisted of the following modules:

- Demographic questions with a sexologist background—this module was required only at the baseline.
- Polish version of the Female Sexual Function Index (FSFI). The questionnaire was independently translated and validated on the Polish population [18,19].
- Official Polish version of the European Organization for Research and Treatment of Cancer Quality of Life Questionnaire of Cancer Patients with the module Cervix-24 (EORTC QLQ-C30 + CX24) [20,21].

At the time of recruitment, each patient received an individual unique code which was used afterwards to complete questionnaires without exposing personal information as the questions were of an intimate nature. Patients filled in the questionnaires online or on paper during control visits. In the case of online questionnaires, patients were contacted at least three times before being considered unavailable. This did not prevent attempting contact at later observation points if there were any left.

Analysis of the results was performed separately for the study groups both with inclusion and exclusion of sexually inactive patients, as sexually inactive patients naturally receive very low scores on the FSFI scale, with “0” points in all of the subscales except the desire subscale. Such analysis was performed to avoid creating bias, as the reasons for sexual inactivity may vary from clinically insignificant to very severe.

Statistical analysis was performed in Statistica 13. The dichotomous variables were compared using the chi-squared test or Fisher’s exact test as required. The continuous variables were compared using Student’s *t*-test or the Mann–Whitney U test as required in the case of unmatched samples. In the case of matched samples, the Wilcoxon signed-rank test was performed. In the case of more than two analyzed groups, the Kruskal–Wallis ANOVA analysis was performed as required. In the case of multiple matched measurements comparison, the ANOVA analysis for matched measurements or Friedman ANOVA analysis with Kendall coefficient for concordance was used as required. A *p*-value of less than 0.05 was considered significant.

## 3. Results

The study group consisted of 35 patients who underwent LEEP and 44 patients who underwent surgical conization of the cervix. Patients in the study group were recruited at the time of qualification for the surgery. The follow-up ratio was as follows:

- At three months of observation: 32 out of 35 patients in the LEEP group (91.42%) and 39 out of 44 patients in the surgical conization group (88.63%).
- At six months of observation: 29 out of 35 patients in the LEEP group (82.86%) and 37 out of 44 patients in the surgical conization group (84.09%).
- At twelve months of observation: 28 out of 35 patients in the LEEP group (80%) and 35 out of 44 patients in the surgical conization group (79.55%).

The baseline characteristics of the study group are presented in Table 1. No significant differences were observed between the LEEP and the surgical conization groups.

There were no significant differences in the rate of women who were sexually active at each time point of observation. No significant differences were observed between both study groups. The detailed results are presented in Table 2.

Among the whole group of patients who underwent LEEP, a statistically significant deterioration in the orgasm subscale was observed. Significant differences were observed in the post hoc analysis between the baseline and both the three- and six-month points of observation. No significant differences were observed in other subscales or the general FSFI score. In the subgroup of sexually active patients, no significant differences were observed in any subscale or the general FSFI score. The detailed results are presented in Table 3. In the LEEP group, there was a significant decrease in the orgasm score. However, this effect was more prominent at 3 and 6 months and became less visible after 12 months of observation (Table 3). This suggests that the effect on orgasm satisfaction was transient and diminished over time.

In the general group of patients who underwent surgical conization, significant differences were observed in the arousal and orgasm subscales, as well as in the general FSFI score. In the post hoc analysis, respective significant differences were observed: for the arousal subscale, between baseline and three months of observation; for the orgasm subscale, between three and twelve months of observation; for the general FSFI score, between baseline and three months of observation. Similar observations were made among sexually active patients. Significant changes were observed in the arousal and orgasms subscales as well as in the general FSFI score. In the post hoc analysis, significant differences were observed as follows: for the arousal subscale, between the baseline and all other observation points; for the orgasm subscale, between baseline and six months of observation and between six and twelve months of observation; for the general FSFI score, between baseline and three months of observation. Detailed results are presented in Table 4.

Upon comparing the baseline and 12-month data for patients undergoing surgical conization (Table 4), we observed that the effects on arousal, orgasm, and total sexual function were transient. Arousal scores decreased significantly at 3 and 6 months, but by 12 months, the score had returned to baseline levels, indicating a temporary effect. Orgasm scores also decreased at 3 and 6 months, but by 12 months, the score was similar to baseline, reflecting that the improvement in orgasm satisfaction was short-lived. The total FSFI score followed a similar pattern, with a decrease at earlier time points that was not sustained at 12 months.

No significant differences were observed in the EORTC QLQ30 CX24 module among the LEEP group. A significant difference was observed in the sexual activity subscale in the group of patients who had undergone surgical conization. In the post hoc analysis, significant differences (deterioration) were observed between baseline and three months of observation. Detailed results are presented in Table 5 and Table 6.

## 4. Discussion

The results obtained in the presented study in the LEEP group are similar to other studies on the subject. The only statistically significant difference we observed was in the orgasm subscale in the whole group—patients after LEEP had significantly lower scores in this subscale after three and six months. There was no such observation in the subgroup of sexually active patients.

The study by Inna N. et al., which analyzed 89 patients after LEEP, with a mean time of observation of 29 weeks after the intervention, revealed a significant but mild decrease in sexual satisfaction, satisfaction with orgasm, and vaginal elasticity [16]. The tools used by the authors were different from ours, and therefore, we cannot relate to vaginal elasticity, but the decrease in the satisfaction from orgasm is coherent with our study.

Serati M. et al. presented a group of 58 patients who had undergone LEEP and were observed 6 months post-intervention [12]. They used the same tool—FSFI—as in our study. The authors observed a significant decrease in the desire subscale. No significant differences were demonstrated in the orgasm subscale. Even though their findings may initially seem different from ours, after more thorough analysis both are associated with psychological aspects of sexuality and not physical, such as lubrication and pain.

The study of Heinzler J. et al. revealed significant deterioration in the level of sexual anxiety of patients who had undergone LEEP [14]. The multivariate analysis performed by the authors showed no significant differences in the physical aspects of sexuality despite the continuous presence of increased sexual anxiety. The study of Hellsten C. et al. in a group of 45 patients who underwent LEEP showed a significant deterioration in the frequency of sexual intercourse, sexual interest, and arousal after 6 months of observation [15]. The deterioration was not temporary—it was still present in the aspects of sexual intercourse frequency and sexual interest two years after the procedure.

The study by Kim B.R. et al. analyzing the effect of LEEP on sexual function also used the FSFI and showed no significant differences after six to twelve months post-procedure. However, this study was performed on a group of only 24 patients, and therefore, this observation should be treated with adequate caution [22].

The only study presenting a beneficial effect of LEEP on sexual function was published by Sadoun C. et al. [13]. At three months post-intervention, patients functioned significantly better in the aspects of desire and orgasm.

The analysis of the literature and our results does not lead to clear conclusions. Despite the relatively low level of surgical invasiveness, LEEP seems to have a negative and not necessarily transient impact on female sexual function. Some theoretical studies suggest that the use of electrical diathermy may lead to excessive scarification and therefore cause dyspareunia [23,24]. However, this does not correspond with our results and data found in the literature on the subject.

Both Hellsten and Heinzler accordingly observed patients with suspected cervical dysplasia who did not require any intervention after the colposcopy [14,15]. Patients who had undergone colposcopy presented similar deterioration in sexual function as those who required LEEP—the effect of LEEP was minimal, if even present. Their results suggested that the negative impact of LEEP may be purely psychological and result uniquely from the diagnosis of dysplasia, not from the intervention itself.

The deterioration of sexual function was more visible in the surgical conization group. A significant decrease was observed in the general group in both arousal and orgasm subscales, which led to a statistically significant decrease in the general FSFI score, with similar results in the sexually active subgroup. However, our results are contradictory to those presented in the literature.

The first original study on the subject published by Kilkku P. et al. in 1982 showed a potential positive effect of surgical conization on female sexual function; after six and twelve months post-surgery, patients reported symptoms of dyspareunia less often than before the intervention [9]. Another cross-sectional study from 2012 by Song T. et al. analyzed the sexual function of patients after surgical conization with a mean observation time of 30 months [8]. They observed no significant changes in female sexual function.

Sparic R. et al. analyzed the long-term effects of surgical conization on female sexual function [10]. The mean observation time was 4.8 years, and no changes in sexual function after the intervention were observed. However, their study had important limitations: the analysis was performed in a combined group of patients after LEEP and surgical conization, questions were not standardized, and there was substantial long-observation recall bias.

Even though our results primarily seem to be contradictory to those in the literature, we observed a significant but transient decrease in sexual function; changes in both the orgasm and arousal subscales, as well as in the general FSFI scores, were observed after three and six months and resolved after twelve months. The studies mentioned above were characterized by long observation periods—30 months in Song’s study, 4.8 years in Sparic’s study, and 6/12 months in Kilkku’s study [9,10]. Therefore, the transient deterioration of female sexual function observed in our study could be overlooked by other authors due to differences in the follow-up time points. Additionally, all studies except for the study by Kilkku P. et al. [9] had possible recall bias due to their cross-sectional nature. Although it was prospective, patients were asked about their sexual function just before the surgery, which could create an illusion of improvement after the surgery. The influence of the time point before surgery might have augmented the level of sexual anxiety and dyspareunia symptoms. Therefore, the observed improvement in this area could have been a relative return to a normal state.

One of the limitations of our study was a relatively small study group, which consisted initially of 79 patients divided into two subgroups of 35 and 44 women, out of which 63 were followed up until the last point of observation. Secondly, despite the prospective nature of the study, the recall bias of the patients at the baseline could affect the results. The sole diagnosis of HPV-related disease such as cervical dysplasia could have a negative effect on female sexuality and sexual function. Another limitation of our study included lack of information about desire for pregnancy and the premenopausal/menopausal status.

The study’s strengths included separate results analysis depending on the type of procedure—surgical conization or LEEP—as well as collection and comparison of patient-reported sexual outcomes in a prospective observation at the baseline and after three study time points.

Future studies on a similar topic could potentially omit the limitations of our study; the patients could be recruited at the beginning of the diagnostic process, and the analyzed group could be significantly larger.

## 5. Conclusions

Our findings indicate that both procedures have a mild, transient negative impact on female sexual function, which tends to resolve within one year. The lack of significant long-term differences in sexual function between the two groups suggests that both procedures have similar consequences on female sexual health. However, larger studies are needed to confirm these results and provide more definitive conclusions.

## Figures and Tables

**Table 1:** Baseline characteristics of the groups.

Feature	LEEP (n = 35)	Surgical Conization (n = 44)	*p*
Age (±SD) [years]	36.03 ± 8.14	36.36 ± 8.96	0.99
Number of pregnancies (median; quartiles)	1 (0–2)	1 (0–2)	0.93
Number of labors (median; quartiles)	1 (0–2)	1 (0–2)	0.95
Age of sexual initiation (mean ± SD) [years]	18.49 ± 2.77	18.13 ± 2.72	0.87
Number of sexual partners (median; quartiles)	4 (2–6)	3 (2–5)	0.37
Higher education (%)	20 (57.14)	24 (54.55)	0.72
Living in a city of 100,000 or more inhabitants (%)	19 (54.29)	24 (54.55)	0.74
Married (%)	15 (42.86)	22 (50.0)	0.62
Employed (%)	24 (70.59)	30 (69.77)	0.92
Post-menopausal (%)	4 (11.76)	6 (13.95)	0.96
Smoking (%)	8 (23.53)	11 (25.58)	0.14
Non-heterosexual orientation (%)	4 (11.43)	4 (10.0)	0.49
Stable sexual partner (%)	30 (88.24)	33 (75.0)	0.51
Baseline FSFI score (all patients; median; quartiles)	27.9 (18.1–31.0)	27.1 (2.4–31.1)	0.44
Baseline FSFI score (sexually active patients; median; quartiles)	28.6 (23.9–31.10)	29.3 (24.6–32.4)	0.10

**Table 2:** Sexual activity during observation.

Group (n, %)	At Baseline	After 3 Months	After 6 Months	After 12 Months	*p* (Multiple Comparisons)
LEEP	28 (80.0%)	24 (70.6%)	21 (72.4%)	19 (67.9%)	0.52
Cervical conization	33 (75.0%)	29 (74.4%)	30 (81.1%)	29 (82.9%)	0.75
In total	61 (77.2%)	53 (72.6%)	51 (77.3%)	45 (72.6%)	0.85

**Table 3:** FSFI results in the LEEP group.

Subscale (Mean ± SD)	At Baseline	After 3 Months	After 6 Months	After 12 Months	*p* (Multiple Comparisons)
Desire	3.53 ± 1.22	3.62 ± 1.38	3.87 ± 1.17	3.99 ± 1.04	0.14
Desire (sexually active)	3.84 ± 1.15	4.03 ± 1.23	4.20 ± 0.97	4.23 ± 0.84	0.89
Arousal	3.83 ± 2.02	3.32 ± 2.25	3.36 ± 2.32	3.42 ± 2.38	0.36
Arousal (sexually active)	4.46 ± 1.36	4.43 ± 1.31	4.71 ± 0.99	4.83 ± 0.88	0.14
Lubrication	4.08 ± 2.10	3.72 ± 2.35	3.73 ± 2.43	3.64 ± 2.50	0.92
Lubrication (sexually active)	4.71 ± 1.37	4.96 ± 1.02	5.16 ± 0.74	5.12 ± 1.03	0.40
Orgasm	**3.98 ± 2.08**	**3.19 ± 2.29**	**3.09 ± 2.24**	**3.39 ± 2.44**	**0.02**
Orgasm (sexually active)	4.57 ± 1.39	4.25 ± 1.54	4.27 ± 1.33	4.82 ± 1.29	0.26
Satisfaction	3.95 ± 2.08	3.65 ± 2.35	3.61 ± 2.44	3.50 ± 2.50	0.83
Satisfaction (sexually active)	4.57 ± 1.41	4.92 ± 1.00	5.06 ± 0.87	4.93 ± 1.16	0.94
Pain	3.69 ± 2.27	3.30 ± 2.35	3.27 ± 2.37	3.27 ± 2.46	0.77
Pain (sexually active)	4.40 ± 1.66	4.40 ± 1.56	4.52 ± 1.40	4.59 ± 1.54	0.82
Total	23.06 ± 10.55	20.61 ± 12.08	20.86 ± 12.01	21.04 ± 12.30	0.40
Total (sexually active)	26.55 ± 6.53	26.94 ± 5.98	28.0 ± 4.06	28.53 ± 4.52	0.64

**Table 4:** FSFI results in the surgical conization group.

Subscale (Mean ± SD)	At Baseline	After 3 Months	After 6 Months	After 12 Months	*p* (Multiple Comparisons)
Desire	3.63 ± 1.27	3.62 ± 1.15	3.87 ± 1.01	3.89 ± 1.00	0.62
Desire (sexually active)	4.23 ± 0.82	4.15 ± 0.81	4.15 ± 0.83	4.20 ± 0.77	0.69
Arousal	**3.69 ± 2.14**	**3.01 ± 2.02**	**3.29 ± 2.02**	**3.53 ± 2.10**	**0.001**
Arousal (sexually active)	**4.78 ± 0.82**	**4.05 ± 1.18**	**4.05 ± 1.40**	**4.34 ± 1.42**	**0.001**
Lubrication	4. 11 ± 2.44	3.79 ± 2.58	3.97 ± 2.51	3.95 ± 2.50	0.77
Lubrication (sexually active)	5.35 ± 0.91	5.18 ± 1.37	4.94 ± 1.71	4.90 ± 1.70	0.21
Orgasm	**3.51 ± 2.20**	**3.05 ± 2.22**	**3.29 ± 2.13**	**3.63 ± 2.29**	**0.003**
Orgasm (sexually active)	**4.55 ± 1. 12**	**4.15 ± 1.47**	**4.13 ± 1.53**	**4.55 ± 1.52**	**0.003**
Satisfaction	3.76 ± 2.35	3.50 ± 2.31	3.76 ± 2.28	3.66 ± 2.24	0.36
Satisfaction (sexually active)	5.00 ± 1.06	4.62 ± 1.33	4.52 ± 1.63	4.42 ± 1.62	0.22
Pain	3.91 ± 2.27	3.46 ± 2.36	3.79 ± 2.36	3.83 ± 2.37	0.11
Pain (sexually active)	5.08 ± 0.79	4,70 ± 1.36	4.70 ± 1.63	4.77 ± 1.60	0.21
Total	**22.37 ± 12.38**	**20.82 ± 12.02**	**22.38 ± 11.69**	**22.97 ± 11.78**	**0.003**
Total (sexually active)	**28.98 ± 4.39**	**26.84 ± 6.36**	**26.49 ± 7.87**	**27.17 ± 7.75**	**0.001**

**Table 5:** EORTC QLQ CX24 sexual subscales in the LEEP group.

Index (Mean ± SD)	At Baseline	After 3 Months	After 6 Months	After 12 Months	*p* (Multiple Comparisons)
Anxiety	24.69 ± 23.74	29.63 ± 31.12	24.69 ± 27.09	28.39 ± 30.24	0.34
Activity	48.15 ± 33.76	41.97 ± 32.81	53.09 ± 37.29	45.68 ± 35.98	0.22
Pleasure	66.90 ± 30.59	73.33 ± 27.78	71.67 ± 27.09	70.00 ± 28.41	0.52
Vaginal function	13.33 ± 17.19	14.17± 14.59	14.17 ± 14.83	14.58 ± 14.78	0.94

**Table 6:** EORTC QLQ CX24 sexual subscales in the surgical conization group.

Index (Mean ± SD)	At Baseline	After 3 Months	After 6 Months	After 12 Months	*p* (Multiple Comparisons)
Anxiety	12.64 ± 16.46	17.24 ± 19.14	16.09 ± 19.16	18.68 ± 20.70	0.28
Activity	**49.42 ± 36.12**	**39.09 ± 36.81**	**45.98 ± 33.82**	**48.28 ± 32.84**	**0.03**
Pleasure	66.50 ± 33.33	65.21 ± 29.26	68.12 ± 27.48	68.12 ± 29.26	0.72
Vaginal function	10.61 ± 13.41	10.98 ± 9.41	12.88 ± 13.04	10.23 ± 9.94	0.84

## Data Availability

Datasets are available from the corresponding author upon request.
